# Urinary incontinence in systemic sclerosis: a prospective multicentre cohort study

**DOI:** 10.1007/s00296-022-05178-1

**Published:** 2022-08-09

**Authors:** Gregor John, Elisabetta Zanatta, Pamela Polito, Silvia Piantoni, Micaela Fredi, Yann Coattrenec, Romain Guemara, Franco Franceschini, Marie-Elise Truchetet, Franco Cozzi, Paolo Airò, Carlo Chizzolini

**Affiliations:** 1 Department of Internal Medicine, Neuchâtel Hospital Network, Rue de la Maladière 45, CH-2000 Neuchâtel, Switzerland; 2 Department of Medicine, Rheumatology Unit, University Hospital of Padova, 35121 Padova, Italy; 3 Rheumatology and Clinical Immunology Unit, Department of Clinical and Experimental Sciences, ASST Spedali Civili and University of Brescia, 25123 Brescia, Italy; 4 Department of Pathology and Immunology, University Medical Centre, Geneva University, Rue Michel-Servet 1, CH-1211 Geneva, Switzerland; 5 Department of Rheumatology, Bordeaux University and Bordeaux Hospital, Bordeaux, France; 6 Department of Medicine, Geneva University, Michel-Servet 1, 1206 Geneva, Switzerland

**Keywords:** Scleroderma, Urinary incontinence, Quality of life, Death, Hospital admission, Natural history

## Abstract

Investigate the natural history of urinary incontinence (UI) in systemic sclerosis (SSc) and assess its impact on quality of life (QoL). A longitudinal, international observational study followed 189 patients with SSc for a median duration of 5 years (IQR: 4.8–5.3). Presence, subtype and severity of UI, hospital admission and QoL were assessed using serial self-administered questionnaires. Mortality data came from national death registries. Multilevel mixed-effect logistic regressions explored factors associated with UI. Cox models adjusted the effects of UI on hospitalization and death for age, sex and subtype of SSc. Mean annual rates of new-onset UI and remission were 16.3% (95%CI 8.3%–24.2%) and 20.8% (95%CI 12.6–29.1), respectively. Among UI patients, 57.9% (95%CI 51.8–64.0) changed from one UI subtype to another. Between annual questionnaires, the severity of UI was the same in 51.1% (95%CI 40.8–61.4), milder or resolved in 35.2% (95%CI 25.3–44.9), and worse in 13.8% (95%CI 6.7–20.9). Anti-centromere antibodies, digestive symptoms, sex, age, neurological or urological comorbidities, diuretics and puffy fingers were all associated with UI. The two strongest predictors of UI and UI subtypes were a recent UI episode and the subtype of previous leakage episodes. UI at inclusion was not associated with hospital admission (adjusted HR: 1.86; 95%CI 0.88–3.93), time to death (aHR: 0.84; 95%CI 0.41–1.73) or change in QoL over time. Self-reported UI among SSc patients is highly dynamic: it waxes and wanes, changing from one subtype to another over time.

**Supplementary Information:**

The online version contains supplementary material available at 10.1007/s00296-022-05178-1.

## Introduction

Urinary incontinence (UI) affects the quality of life (QoL) [1] and work productivity [2], increases the risks of falls [3], mood disorders [4, 5] and institutionalisation [6], and it has been associated with higher mortality [7, 8]. Thus, UI has a significant impact on individuals and their medical care [9].

UI is now recognised as a highly prevalent condition in cases of systemic sclerosis (SSc), affecting 52–63% of patients [10, 11]. Epidemiology and associated factors are very different to those of UI in the general population or in other conditions [10]. Thus, its pathogenesis possibly involves SSc-specific mechanisms that have yet to be fully elucidated [12]. Fibrosis and vascular anomalies have been reported in the bladder of SSc patients [13–18]. However, an autopsy study found no more fibrosis in the bladders of sclerodermic patients, than in controls [19]. Alteration of the nervous system, notably dysfunction of the parasympathetic system seen in SSc, could play a role in UI [16, 18]. But Minervini et al. failed to link autonomic function and urinary symptoms [16]. Menopause can appear earlier in SSc and might play a role in women [20]. Finally, an antibody-mediated mechanism is supported by in vitro analysis [21], and parallels made with other rheumatologic diseases [22].

To date, there were no data available on the natural history of UI in SSc. Some reports have found a higher prevalence of UI in the limited form of SSc or among patients having anti-centromere antibodies (ACA) [11], whereas others have found an association with anti-topoisomerase I (anti-Scl70) antibodies [10], with a very different prognosis [23]. Thus, we aimed to investigate the natural history of UI in SSc, the factors influencing it and explore UI as an indicator of unfavourable outcomes by focusing on QoL, physical well-being and the risks of hospital admission or death.

## Patients and methods

### Study overview

This international, prospective, observational study enrolled patients suffering from SSc at four European tertiary hospital centres from January 2013 to December 2015. Patients underwent three annual evaluations of their lower urinary tract symptoms (LUTS) by questionnaires in two centers, two annual evaluations in one center and one evaluation in the last center. All patients were then followed until death or to 31 December 2019. The study complies with the Declaration of Helsinki. Locally appointed ethics committee has approved the research protocol. Written informed consent has been obtained from all patients included in the study. The following sections summarise our cohort population data, inclusion process and variable management, which have been published previously [11, 24].

### Study outcomes

The main outcome was continence evolution at annual clinical evaluations. The secondary outcomes were first UI episode, QoL, first hospital admission, number of admission, length of stay, and death.

Scenarios of urinary continence evolution between study visits were “new-onset UI”, “resolved UI symptoms”, “worsening of UI symptoms”, “decreasing UI symptoms”, and “changing UI subtype”. The first described patients continent of urine in the previous study visit, who complained of UI in the follow-up visit. The second described patients continent of urine, who used to complain of UI in the last study visit. The three last scenarios refer to patients with UI in two study visits, who changed the frequency of UI episodes (from daily to monthly for example), or changed the subtype of UI (changing from stress to urge UI for example).

### Study population

Eligible patients were aged 18 or older and satisfied the ACR/EULAR 2013 SSc criteria [25]. They were included consecutively at each participating centre. Those unable or unwilling to follow the study’s rules, end-of-life patients, pregnant women and anuric patients were excluded.

### Symptoms and measurement

At inclusion and each annual study visit, participants completed a thirty-minute self-administered questionnaire on their LUTS, QoL, functional status, disease activity, medication, medical history and demographic characteristics. Patients were classified as having the limited or diffuse cutaneous form of SSc, according to Le Roy et al. [26].

At each study visit, UI was characterised using the International Consultation on Incontinence Modular Questionnaires (ICIQ-FLUTS and ICIQ-MLUTS) [27]. These explore the last four weeks’ LUTS and use standards recommended by the International Continence Society [28]. Participants could also complete a between-visits form for new episodes of UI occurring between study visits. UI was defined as any involuntary leakage of urine and was subdivided into stress (SUI), urge (UUI) and mixed UI. Leakage severity was further stratified according to leakage frequency (monthly, weekly or daily leakage).

The 36-item short-form health survey (SF-36) [29, 30] was summarised into its physical component (PCS) and mental component (MCS), and the Incontinence Quality of Life (I-QOL) questionnaire was provided as a single, transformed scale ranging from 0 to 100 points [31]. Functional activities were explored using the Scleroderma modified Health Assessment Questionnaire and Disability Index (SSc-HAQ-DI) and the Cochin scale [32].

First hospital admission, length of stay and number of admissions between study visits were collected using self-administered questionnaires and participating hospitals’ electronic databases. Data on the date of death were obtained from national death registries and each participating hospital’s electronic databases until the end of December 2019. Each participating centre stratified the cause of death into SSc-related and SSc-unrelated based on their expertise.

### Statistics

The initial sample involved five institutions and was calculated to demonstrate differences in UI prevalence between SSc subtypes at the baseline evaluation [11]. However, only four of the five institutions took part in the prospective cohort, which was therefore composed of 189 SSc individuals.

Comparisons between the continent and incontinent participants were performed using the appropriate chi-squared test or Fischer’s exact test for categorical variables. The two-sided Mann–Whitney test was used for continuous variables. Variables for the same individual measured on two occasions (QoL, HAQ-DI and Cochin scale) were compared using the paired Wilcoxon signed-rank test.

To evaluate factors associated with the natural history of UI, we calculated multilevel mixed-effect logistic regressions accounting for repeated measures and patients being clustered in different study centres. Analyses were repeated for any evolutions in UI scenarios at annual clinical evaluations. A random intercept was given to any individual to account for repeated measures. Other models explored risks by UI subtype and severity (dependent variables). Potential associated factors were chosen based on our previous publications [11, 24]. Considering the small sample size and the number of events, we performed exploratory univariate analyses and decided not to perform multivariate analyses.

The unadjusted effects of UI on time-dependent outcomes (first UI episode, first hospital admission or death) were analysed using Kaplan–Meier survival analysis and unweighted, two-sided, log-rank tests to compare groups. Participants with unknown status on 31 December 2019 were considered lost to follow-up and were censored from their last medical visit. Multivariate Cox models were used to adjust for age, sex and type of SSc. The same analyses were performed according to the subtype and severity of UI and SSc-related death.

The significance level was set at 5%, and all analyses were performed using STATA statistical software, version 12.0 (StataCorp LP, College Station, TX, USA).

## Results

**Fig. 1:**
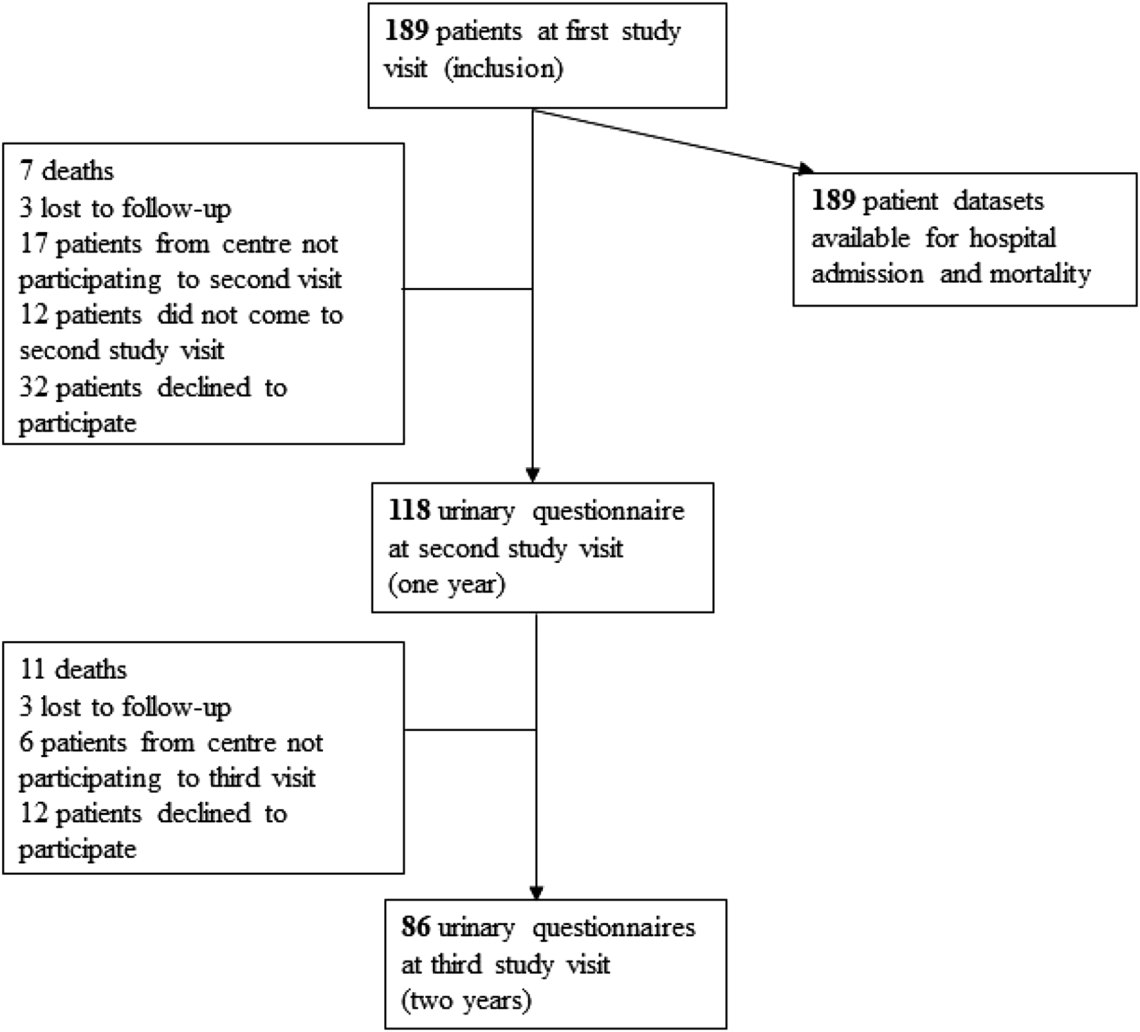
Study flowchart

A total of 189 SSc patients were followed for a median duration of 5 years (IQR: 4.8–5.3) (Fig. 1). Their second and third study visits were at a median of 1.1 (IQR: 1.0–1.1) and 2.1 (IQR: 2.0–2.7) years after inclusion, respectively. Patients’ principal characteristics are given in Table 1.

**Table 1:** Main characteristics and differences between groups with or without urinary incontinence at inclusion

	Urinary incontinence			
	Entire cohort (189)	Present (111)	Absent (78)	*P* value
General				
Age, median y (IQR)	60 (50–69)	62 (51.5–70.5)	58.5 (47–66)	0.057
Men, n (%)	20 (10.7)	5 (4.6)	15 (19.2)	0.002
Body mass index	23.0 (20.5–26.3)	23.3 (20.3–27.7)	22.9 (20.7–25.3)	0.520
Smoking status				
Current	14 (7.5)	8 (7.4)	6 (7.7)	0.311
Former	65 (34.9)	33 (30.6)	32 (41.0)	
Never	107 (57.5)	67 (62.0)	40 (51.3)	
Children	2 (1–2)	2 (1–2)	2 (1–2)	0.978
Birth–natural route	1 (0–2)	1 (0–2)	1 (0–2)	0.943
Comorbid conditions				
Diabetes	9 (5.0)	6 (5.7)	3 (4.1)	0.641
Heart disease	23 (13.1)	14 (13.3)	9 (12.7)	0.891
Pulmonary disease	71 (39.4)	41 (38.3)	30 (41.1)	0.708
Neurological palsy*	4 (2.2)	3 (2.8)	1 (1.4)	0.650
Urological/ gynaecological disease^†^	9 (5.0)	6 (5.7)	3 (4.1)	0.461
Medication				
Corticoids	78 (41.9)	41 (38.0)	37 (47.4)	0.196
Diuretics	45 (24.2)	30 (27.8)	15 (19.3)	0.178
Opioids	15 (8.1)	6 (5.6)	9 (11.5)	0.139
Side effects^‡^	14 (7.6)	9 (8.3)	5 (6.5)	0.781
Systemic sclerosis				
Diffuse cutaneous SSc	63 (33.9)	27 (25.0)	36 (46.1)	0.003
Limited cutaneous SSc	123 (66.1)	81 (75.0)	42 (53.8)	0.003
Anti-nuclear antibodies	179 (98.4)	102 (98.1)	77 (98.7)	0.737
ACA	65 (35.5)	46 (43.8)	19 (24.4)	0.007
Scl70 antibodies	70 (38.3)	38 (36.2)	32 (41.3)	0.506
Disease duration (y)	11.8 (6.7–18.9)	12.6 (6.7–19.8)	10.8 (6.3–17.5)	0.385
Age at first Raynaud (y)	42.5 (30.7–52.4)	43.2 (33.4–52.6)	41.0 (28.2–52.4)	0.465
MRSS	4 (2–10)	4 (2–9)	6 (2–12)	0.078
Digestive symptoms	142 (77.2)	88 (83.2)	54 (69.2)	0.028
Digital ulceration	97 (53.3)	47 (45.2)	50 (64.1)	0.011
Finger-skin thickening	94 (51.7)	59 (56.2)	35 (45.5)	0.152
Synovitis	15 (8.2)	9 (8.5)	6 (7.7)	0.845
Lung fibrosis	49 (27.6)	29 (27.1)	20 (27.8)	0.921
Pulmonary hypertension	18 (10.1)	14 (13.1)	4 (5.6)	0.130
Disability				
HAQ-DI	0.625 (0.125–1.25)	0. 625 (0.25–1.25)	0.375 (0.0–1.125)	0.026
6MWT (m)	380 (315–460)	340 (280–395)	410 (340–525)	0.019

ACA, Anti-centromere antibodies; Scl70, an antibody directed against topoisomerase; HAQ-DI, Health Assessment Questionnaire-Disability Index; y, year; SSc, systemic sclerosis; IQR, interquartile range; MRSS, modified Rodnan skin score; 6MWT, 6-min walking test

*Neurological diseases (central or peripheral) that result in palsy; ^†^ Known urethral stricture, benign prostatic hyperplasia, prostate cancer, prolapse (uterus, rectum or bladder), or bladder cancer in the past; ^‡^ Medical drugs with known urinary side effects (e.g. tricyclic antidepressant, antipsychotic, antiparkinsonians, muscle relaxant, antihistamines, antispasmodic)

### Urinary incontinence status and severity over time

**Fig. 2:**
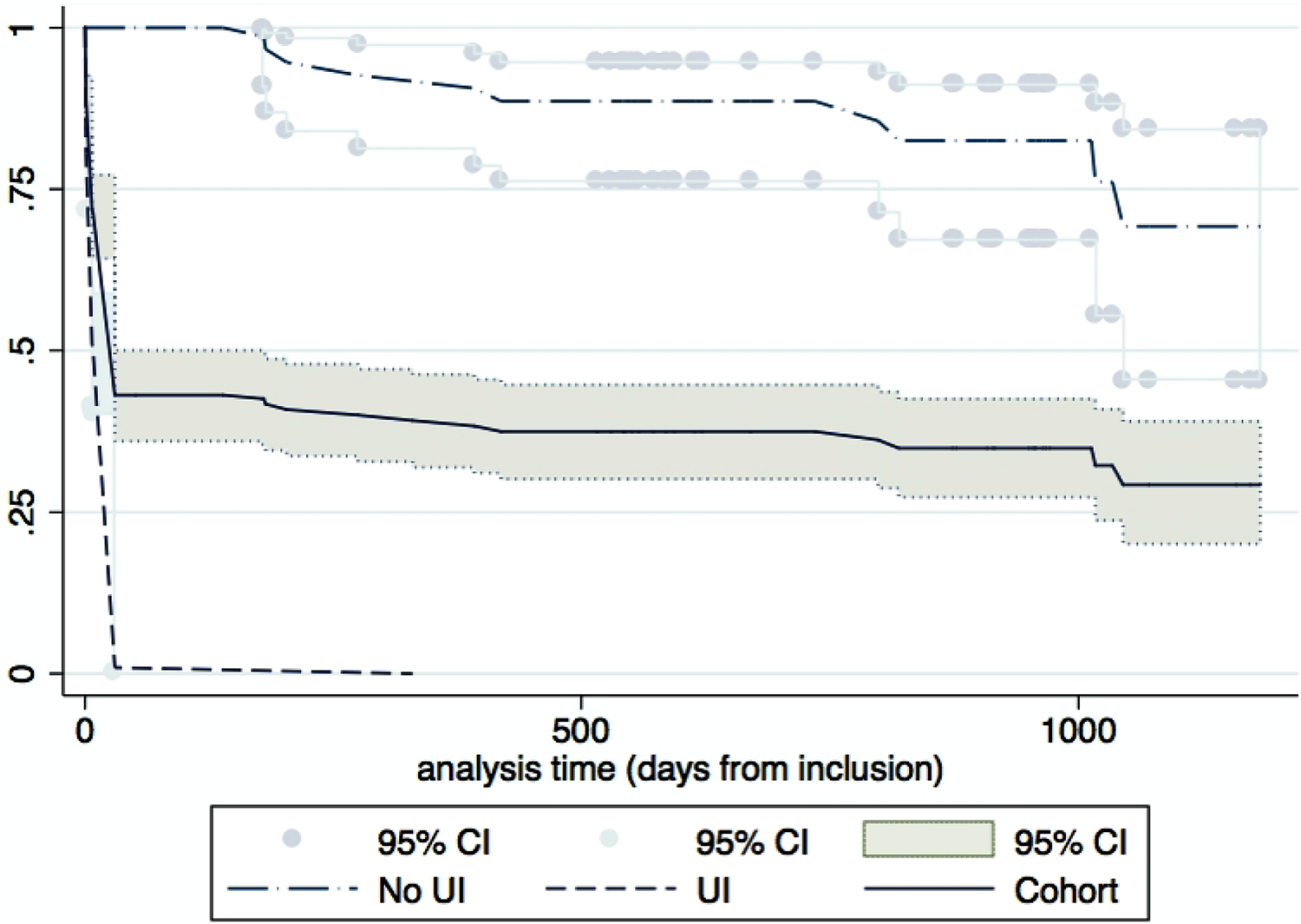
Time to first urinary incontinence episode shown against urinary continence status at admission and for the complete study-cohort. After a steep drop due to patients with UI at inclusion (dash), the curve shows a shallower decrease due to new UI episodes among patients continent of urine at study inclusion (long dash-dot). UI: urinary incontinence

Of 189 patients, 118 were followed for their urinary symptoms for one year and 86 for two years (Fig. 1). The time to first UI episode is presented in Fig. 2. After a steep drop due to patients with UI at inclusion, the curve followed a shallower decrease due to new UI episodes among patients continent of urine at study inclusion.

**Fig. 3:**
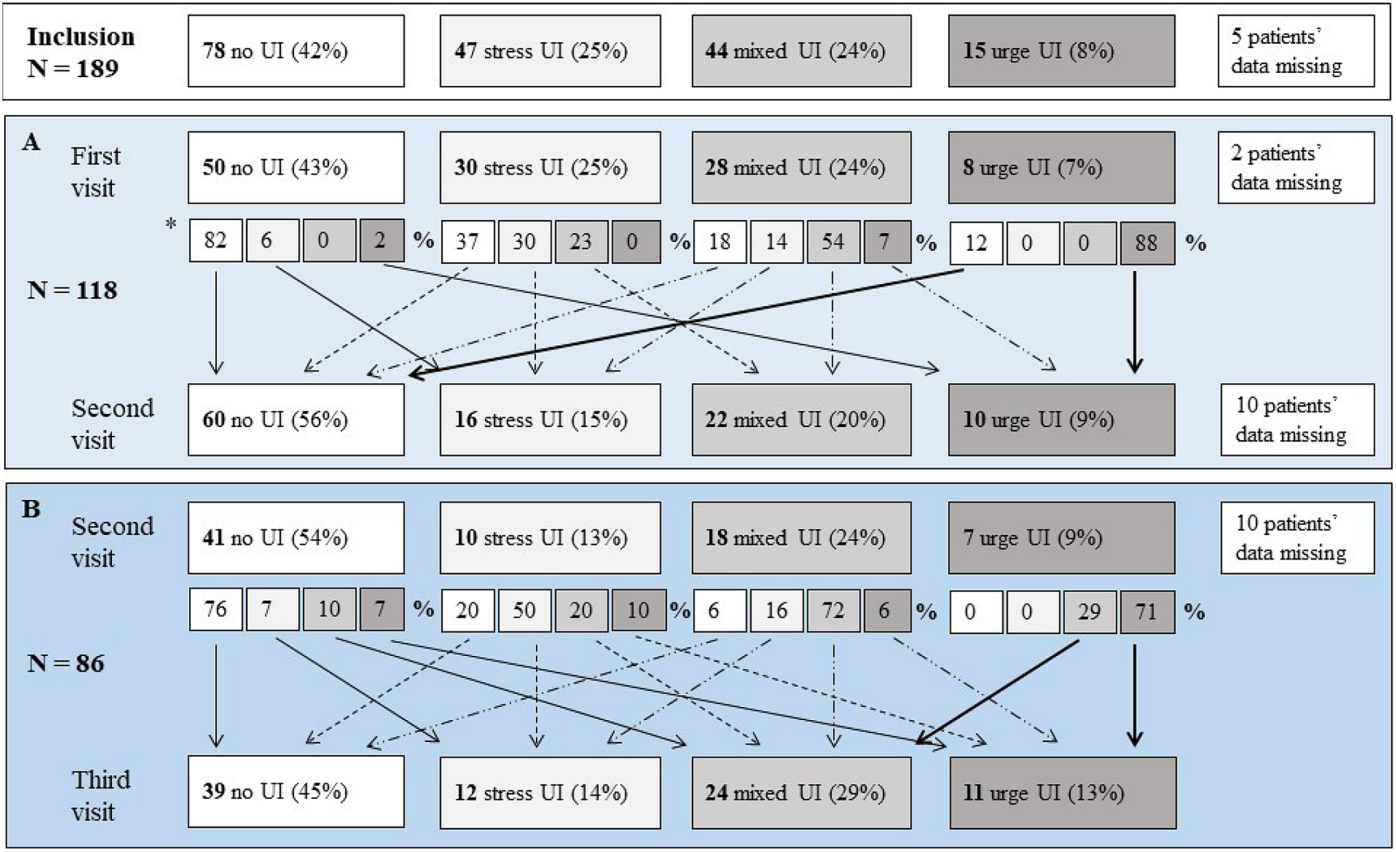
Prevalence and types of urinary incontinence (UI) between first and second study visits (panel **A**) and between second and third study visits (panel **B**). Small boxes and arrows show the percentages of patients whose subtype of UI had changed. * Due to missing data, the sums of the proportions do not equal 100 per cent. UI: urinary incontinence

**Fig. 4:**
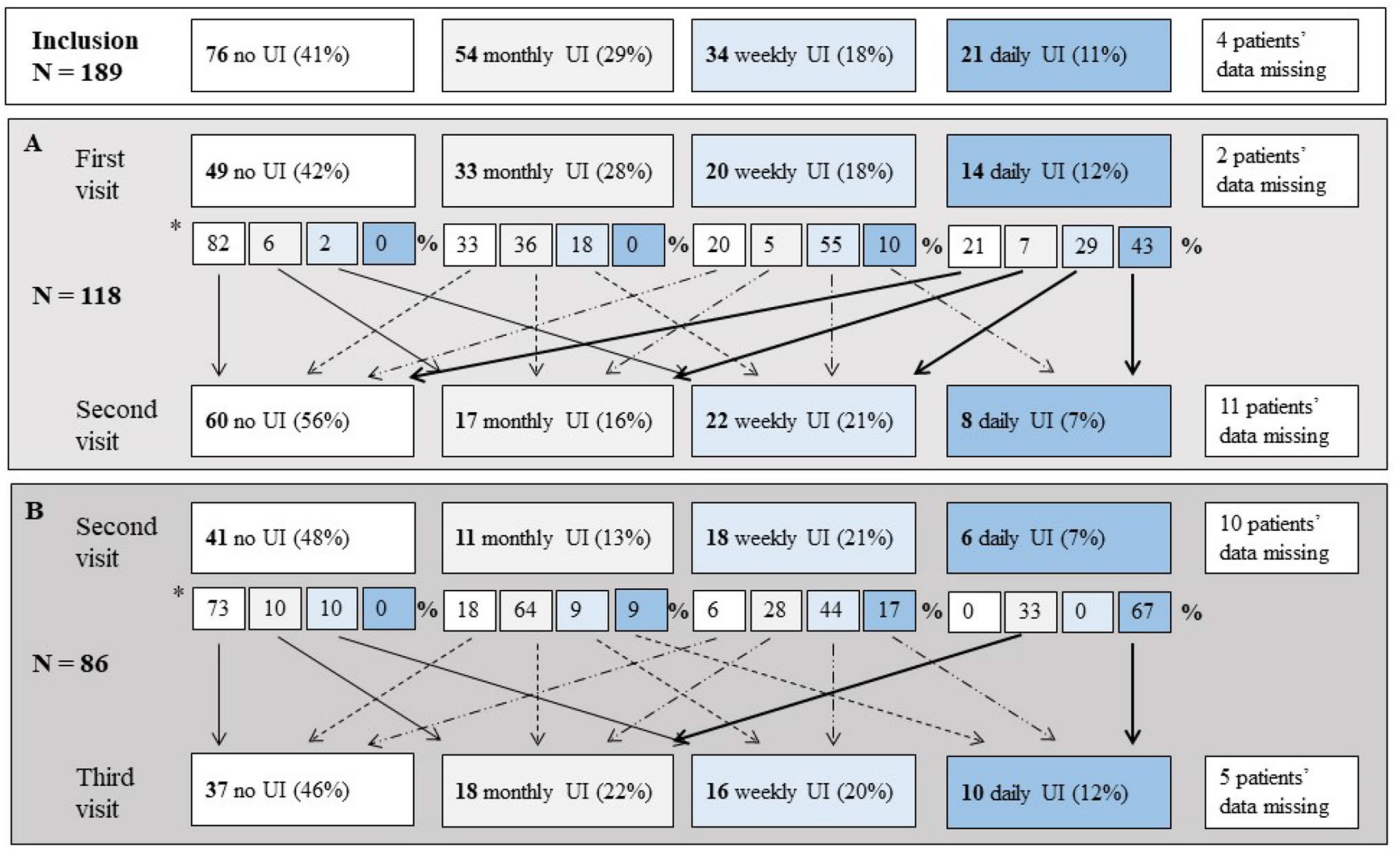
Prevalence and severity of urinary incontinence (UI) between first and second study visits (panel **A**) and between second and third study visits (panel **B**). Small boxes and arrows show the percentages of patients whose severity of UI had changed. * Due to missing data, the sums of the proportions do not equal 100 per cent. UI: urinary incontinence

Continence status changed for 34 (29%) of 118 patients between their inclusion and their second visit; it changed for 22 (26%) of 86 patients between their second and third study visits. The yearly rate of new-onset UI was 16.3% (95% CI 8.3%–24.2%). Each year, the symptoms of 20.8% (95% CI 12.6–29.1) of patients suffering from UI resolved, 57.9% (95% CI 51.8–64.0) changed from one subtype of UI to another (e.g., from stress to urge), and 34.2% (95% CI 28.4–40.1) kept the same UI subtype (Fig. 3). Between annual evaluations, the severity of UI was the same in 51.1% (95% CI 40.8–61.4) of patients, milder or resolved in 35.2% (95% CI 25.3–44.9) and worse in 13.8% (95% CI 6.7–20.9) (Fig. 4).

### Factors associated with the evolution of UI in SSc

Factors associated with the natural history of UI are reported in Supplementary Table S1 and S2. Presenting with puffy fingers on clinical evaluation increased the composite risk of “new-onset UI” or “worsening of UI symptoms” (OR 2.6; 95% CI 1.2–5.9). None of the factors was constantly associated with the risk of either “new-onset UI” or “worsening of UI symptoms”.

The combined chance of being attributed an outcome of “decreasing severity of UI”, “resolving UI symptoms” or “maintaining continence” decreased with previous episodes of SUI (OR 0.2; 95% CI 0.1–0.4) or UUI (OR 0.2; 95% CI 0.1–0.4), the presence of ACA (OR 0.1; 95% CI 0.04–0.5) or digestive symptoms (OR 0.2; 95% CI 0.1–0.9), and it was higher in men (OR 9.2; 95% CI 1.2–73.4). These factors’ effects on the risk of “decreasing severity of UI” or the risk of “resolving UI symptoms” were inconstant (Supplementary Table S1).

The risk of UUI at subsequent visits increased with previous episodes of UUI (OR 18.6; 95% CI 8.5–40.6) or SUI (OR 3.7; 95% CI 1.1–12.9), ACA positivity (OR 16.9; 95% CI 2.5–116), digestive symptoms (OR 32.4; 95% CI 3.3–320), an age above than median (OR 9.5; 95% CI 1.5–59.6) and higher frequencies of episodes of UI (Supplementary Table S2). The risk of SUI at the subsequent visit increased with previous episodes of SUI (OR 19.1; 95% CI 8.8–41.6) or UUI (OR 8.4; 95% CI 2.3–31.3) and with higher frequencies of episodes of UI. However, the risk was lower for men (OR 0.01; 95% CI 0.001–0.35).

Age, neurological palsy, urological comorbidities, UUI, SUI, diuretic treatments, ACA positivity, the limited cutaneous form of SSc and the previous severity of episodes of UI were inconstantly associated with monthly, weekly or daily episodes of UI (Supplementary Table S2).

### Quality of life and disability

Except for the “physical functioning” domain of SF-36, patients’ QoL remained stable from the first to the third study visit, and there were no statistical differences between patients with and without UI. This was also true for changes in disability between these two evaluations and between the continent and incontinent patients (Supplementary Table S3).

### Hospital admission and mortality

Between their inclusion and their third study visit, 43 (36%) patients were hospitalised at least once, with a median length of stay of 8 days (IQR: 5–18). UI at inclusion was not associated with time to first hospital admission in our univariate (HR 1.17; 95% CI 0.63–2.16) or adjusted analyses (HR 1.27; 95% CI 0.67–2.42). This was also true when considering different subtypes of UI and severity (Table 2).

**Table 2:** Univariate and multivariable Cox regressions of risk of hospital admission and death stratified by urinary incontinence (UI), UI severity and UI subtype

	Univariate analysis	Adjusted models		
		By UI (binary)	By UI severity	By UI types
Hospital admission				
UI (any)	1.2 (0.7–2.3)	1.3 (0.7–2.4)	–	–
Monthly UI	1.0 (0.4–2.1)	–	1.1 (0.5–2.5)	–
Weekly UI	1.2 (0.5–2.8)	–	1.4 (0.6–3.4)	–
Daily UI	1.8 (0.7–4.4)	–	2.0 (0.7–5.2)	–
Stress UI	0.9 (0.4–2.3)	–	–	1.1 (0.5–2.6)
Urge UI	1.6 (0.5–4.7)	–	–	1.5 (0.5–4.7)
Mixed UI	1.3 (0.6–2.7)	–	–	1.3 (0.6–2.9)
Age (continuous)	1.0 (1.0–1.1)*	1.0 (1.0–1.1)*	1.0 (1.0–1.1)*	1.0 (1.0–1.1)*
Male sex	0.6 (0.2–1.5)	1.5 (0.5–4.2)	1.8 (0.6–5.5)	1.6 (0.7–4.6)
Limited form of SSc	0.4 (0.2–0.8)*	0.4 (0.2–0.7)*	0.3 (0.2–0.7)*	0.3 (0.2–0.7)*
Death				
UI (any)	1.0 (0.5–1.9)	0.9 (0.5–1.9)	–	–
Monthly UI	0.7 (0.3–1.8)	–	0.7 (0.3–1.9)	–
Weekly UI	0.9 (0.3–2.4)	–	0.9 (0.3–2.6)	–
Daily UI	2.2 (0.9–5.4)	–	1.7 (0.6–4.7)	–
Stress UI	0.9 (0.4–2.2)	–	–	1.0 (0.4–2.5)
Urge UI	2.6 (1.0–6.7)	–	–	2.1 (0.8–5.8)
Mixed UI	0.6 (0.2–1.6)	–	–	0.6 (0.2–1.6)
Age (continuous)	1.0 (1.0–1.1)*	1.0 (1.0–1.1)*	1.0 (1.0–1.1)*	1.0 (1.0–1.1)*
Male sex	0.6 (0.2–1.6)	0.7 (0.2–2.1)	0.9 (0.3–2.6)	0.7 (0.2–2.2)
Limited form of SSc	0.8 (0.4–1.6)	0.7 (0.3–1.5)	0.6 (0.3–1.4)	0.8 (0.3–1.8)

SSc: systemic sclerosis; UI: urinary incontinence

Thirty deaths (19%) occurred during the median follow-up time of 5 years (IQR: 4.8–5.3), of which 22 (73%) were deemed to be SSc-related. UI at baseline was not associated with time to death in either our univariate (HR 0.97; 95% CI 0.49–1.92) or adjusted analyses (HR 0.94; 95% CI 0.46–1.91). There were also no associations between UI severity or UI subtype and death from any cause (Table 2) or SSc-related death (data not shown).

## Discussion

This longitudinal, prospective, international multicentre study demonstrated that self-reported urinary incontinence (UI) among systemic sclerosis (SSc) patients is a highly dynamic phenomenon. Over a period of two years, almost 40% of participants changed their urinary status, and an episode of urine leakage (transient or persistent) affected the majority of patients. Nevertheless, compared to continent patients, UI was not associated with an unfavourable change in QoL or functional status over time or with an increased risk of hospital admission or death in SSc.

In contrast to previous beliefs that UI is a binary and constant disease, there is growing evidence that UI changes over time, with active and inactive phases. In the general population, the annual incidence of new-onset UI is 2%–10% among men and 3%–20% among women, with remission rates of 27%–32% and 3%–12%, respectively [33–35]. Thus, patients may experience periods of incontinence followed by periods of continence [36]. After 50–60 years old, rates of new-onset UI are lower than rates of remission, leading to a higher overall prevalence of UI in older individuals [37]. Although the present study had no control group, incidence rates of UI in SSc patients seemed to be higher than in the general population, notably for younger participants. This higher level of new-onset UI came with the previously observed higher prevalence of UI in SSc patients [10, 11].

Although UI is commonly divided into clinically independent subtypes, about a quarter of SSc patients in the present study changed from one UI subtype to another between annual visits. This has also been observed in longitudinal studies of women without SSc [35, 38]. Furthermore, the treatment of one component of mixed UI (e.g. SUI by medication or surgery) may result in the remission of the other component (UUI) [35]. Thus, the boundary between UI subtypes appears to be narrow.

Due to its dynamic nature, UI must be reassessed often (at least once a year) among SSc patients. This reassessment is important to adapt UI management on its severity and subtype and to look for factors that might influence or predict its evolution. Although results were inconstant across the different natural history scenarios of UI over time, several factors—including ACA antibodies, digestive symptoms and, to a lesser extent, sex, age, neurological palsy, urological comorbidities, diuretic treatments, the limited cutaneous form of SSc and the presence of puffy fingers on clinical evaluation—appeared to be potentially associated with UI. Nevertheless, the two strongest predictors of UI and its subtypes were a recent UI episode and the subtype of the previous leaking episodes (SUI or UUI). Understanding the factors that influence the evolution of UI, and which phases of UI have the most significant health effects, will help to reassure some patients, provide tailored treatments for severe episodes of UI that are unlikely to resolve, and also give prevention based on risk factors.

The present study had some limitations. First, as previously mentioned, because no controls were included, it was impossible to confirm whether SSc patients had higher new-onset or remission rates than the general population. Second, UI diagnoses were based on self-reported questionnaires without objective measures. The ICIQ questionnaires have nevertheless been correlated with objective measures of UI in other studies [39, 40]. Third, except for the corticosteroids, no information on suppressive medication was available in the study. Finally, the sample size was small, and the analysis of each scenario of continence evolution over time was restricted to subgroups (e.g. patients with UI for symptoms resolution). This affected the study’s power to detect influencing factors.

In conclusion, UI is a dynamic condition in SSc that waxes and wanes, eventually changing from one form to another subtype.

## Supplementary Information

Below is the link to the electronic supplementary material.

Supplementary file1 (DOCX 44 KB)

## Data Availability

The database is freely available on request at: gregor.john@rhne.ch.

## Abbreviations

ACA: Anti-centromere antibodies
HAQ-DI: Health Assessment Questionnaire Disability Index
ICIQ-FLUTS: International Conference on Incontinence Questionnaire—Female Lower Urinary Tract Symptoms
ICIQ-MLUTS: International Conference on Incontinence Questionnaire—Male Lower Urinary Tract Symptoms
I-QOL: Incontinence Quality of Life (questionnaire)
lcSSc: Limited cutaneous form of Systemic Sclerosis
LUTS: Lower Urinary Tract Symptoms
Scl70: Antibody directed against topoisomerase I
SF-36: Short Form 36 (questionnaire)
UI: Urinary incontinence
