# Supplementary material for: Urinary incontinence in systemic sclerosis: a prospective multicentre cohort study
Source: Rheumatol Int. 2022 Aug 9;42(12):2141–50. doi: 10.1007/s00296-022-05178-1 (PMC9548477; doi:10.1007/s00296-022-05178-1)
Supplement: Supplementary file 1 — Supplementary file1 (DOCX 44 KB) [file 296_2022_5178_MOESM1_ESM.docx]

**Appendix**

**Supplementary Table S1:** Factors associated with the natural history of UI: **page 2-4**

**Supplementary Table S2:** Factors associated with an incident episode of UI by subtype and severity: **page 5-7**

**Supplementary Table S3:** Quality of life (QoL) and evolution of disability between first and third study visits by the presence of urinary incontinence at inclusion: **page 8-9**

| **Table 1**: Factors associated with different scenario for patients with and without urinary incontinence. Results are odds ratios from mixed-effect logistic regression. | | | | | |
| --- | --- | --- | --- | --- | --- |
|  | **Worse** | | **Better** | | |
|  | **New UI** | **New or worse UI** | **Resolve UI** | **Better or resolve UI** | **Maintain continence, better or resolve UI** |
| Number / total (%) | 16 /194 (8.3%) | 31 /192 (16.1%) | 22 / 117 (18.8%) | 35 / 115 (30.4%) | 112 / 194 (57.7%) |
| General |  |  |  |  |  |
| Age > median | 0.67 (0.21-2.12) | 1.52 (0.65-3.55) | 0.46 (0.18-1.20) | 0.86 (0.37-2.03) | 0.45 (0.13-1.56) |
| Men | 0.59 (0.07-4.81) | 0.54 (0.12-2.46) | 1.08 (0.12-10.20) | 1.56 (0.25-9.75) | 9.18 (1.15-73.40) |
| BMI Q1 vs Q2  Q3  Q4 | 1.14 (0.25-5.15)  0.78 (0.14-4.29)  1.36 (0.33-5.56) | 0.80 (0.23-2.81)  1.11 (0.34-3.63)  1.73 (0.64-4.67) | 0.34 (0.06-1.74)  0.40 (0.10-1.68)  0.65 (0.22-1.96) | 0.60 (0.18-2.07)  0.46 (0.14-1.53)  0.69 (0.26-1.83) | 1.11 (0.24-4.94)  0.38 (0.09-1.72)  0.28 (0.07-1.21) |
| Birth-natural route 0 vs 1  2  3 | 1.67 (0.21-13.33)  3.90 (0.76-19.94)  3.87 (0.59-25.46) | 1.33 (0.31-5.15)  2.35 (0.85-6.60)  2.94 (0.83-10.35) | 1.14 (0.27-4.76)  0.91 (0.28-2.97)  0.50 (0.09-2.79) | 1.71 (0.46-6.34)  1.21 (0.41-3.58)  2.00 (0.56-7.14) | 1.19 (0.23-6.03)  0.51 (0.13-1.96)  0.73 (0.13-4.04) |
| Cesarienne 0 vs 1  2 | 1.20 (0.24-5.96)  - | 0.92 (0.27-3.53)  0.37 (0.05-3.42) | 0.74 (0.15-3.69)  1.62 (0.29-9.14) | 1.04 (0.29-3.70)  1.75 (0.36-8.42) | 1.03 (0.19-5.50)  3.39 (0.41-27.87) |
| Pulmonary disease | 0.77 (0.25-2.35) | 0.93 (0.42-2.08) | 1.50 (0.58-3.89) | 1.30 (0.56-2.99) | 2.00 (0.62-6.46) |
| Lung fibrosis | 1.64 (0.58-4.62) | 1.15 (0.53-2.51) | 1.99 (0.77-5.15) | 1.56 (0.68-3.56) | 2.86 (0.97-8.37) |
| Pulmonary hypertension | 0.56 (0.07-4.55) | 0.62 (0.13-2.81) | 1.51 (0.37-6.11) | 1.34 (0.37-4.93) | 1.06 (0.16-7.05) |
| Cardiac disease | 1.29 (0.34-4.97) | 0.96 (0.31-3.00) | 0.97 (0.25-3.75) | 1.28 (0.39-4.13) | 0.94 (0.20-4.46) |
| Heart failure | 2.65 (0.26-26.20) | 2.58 (0.22-29.41) | NA | 2.26 (0.14-37.28) | 0.40 (0.02-9.70) |
| Diabetes | 2.01 (0.39-10.41) | 1.14 (0.23-5.60) | 0.60 (0.07-5.13) | 1.78 (0.38-8.42) | 0.98 (0.10-9.61) |
| Neurological palsy* | - | 1.27 (0.17-11.80) | NA | 2.36 (0.32-17.50) | 1.26 (0.05-29.86) |
| UI related |  |  |  |  |  |
| Urological disease^†^ | - | 1.30 (0.26-6.44) | 0.91 (0.11-7.46) | 1.56 (0.25-9.75) | 3.08 (0.21-44.35) |
| Previous stress UI | - | 0.81 (0.35-1.87) | 20.5 (1.16-100) | 3.21 (0.76-13.58) | 0.18 (0.09-0.36) |
| Previous urge UI | - | 0.72 (0.28-1.82) | 0.39 (0.14-1.08) | 1.1 (0.49-2.52) | 0.17 (0.08-0.38) |
| Previous monthly UI  Weekly UI  Daily UI | - | 1.19 (0.48-2.96)  0.93 (0.33-2.59)  - | - | - | 0.11 (0.05-0.25)  0.09 (0.04-0.21)  0.22 (0.08-0.60) |
| Previous fecal incontinence | 0.38 (0.05-3.12) | 0.79 (0.22-2.88) | 0.80 (0.21-3.06) | 1.44 (0.50-4.11) | 1.43 (0.30-6.83) |
| Medication |  |  |  |  |  |
| No alcohol vs 1 glass/ day**  2 or more glass/ day | - | 1.67 (0.54-4.49)  0.77 (0.17-3.61) | 0.56 (0.16-2.00)  0.33 (0.04-2.61) | 0.93 (0.34-2.59)  0.75 (0.18-3.09) | 1.00 (0.26-3.94)  1.32 (0.23-7.52) |
| Coffee 0-1cup/d vs 1-2 cup/d  2-3 cup/d  > 3 cup/d | 1.18 (0.26-5.35)  1.22 (0.19-7.90)  - | 0.89 (0.30-2.39)  0.87 (0.23-3.20)  - | 0.81 (0.28-2.36)  1.00 (0.31-3.29)  0.51 (0.06-4.38) | 0.58 (0.20-1.67)  1.93 (0.59-6.37)  1.18 (0.38-12.58) | 1.02 (0.21-4.84)  3.54 (0.45-27.83)  11.50 (0.42-317.12) |
| Corticoids | 0.67 (0.23-1.94) | 0.66 (0.30-1.46) | 1.52 (0.63-3.68) | 1.32 (0.59-2.93) | 1.93 (0.68-5.47) |
| Diuretics | 0.65 (0.17-2.41) | 1.15 (0.47-2.79) | 1.93 (0.78-4.78) | 1.33 (0.55-3.19) | 0.88 (0.26-3.02) |
| Opioids | 0.44 (0.05-3.57) | 1.65 (0.56-4.89) | 0.55 (0.07-4.34) | 0.83 (0.20-3.25) | 1.27 (0.29-5.64) |
| Side effect^‡^ | 2.03 (0.21-19.52) | 1.35 (0.27-6.90) | 2.53 (0.75-8.56) | 1.91 (0.49-7.39) | 1.67 (0.19-14.38) |
| Systemic sclerosis |  |  |  |  |  |
| Disease duration >16y | 1.24 (0.42-3.67) | 0.71 (0.32-1.58) | 0.95 (0.39-2.35) | 1.75 (0.77-3.98) | 2.20 (0.70-6.91) |
| Non-raynaud symptoms > 12y | 2.00 (0.64-6.25) | 0.68 (0.29-1.57) | 0.85 (0.33-2.19) | 1.0 (0.42-2.34) | 1.07 (0.32-3.57) |
| Diffuse cutaneous SSc | 1.36 (0.47-3.93) | 1.27 (0.56-2.92) | 0.94 (0.32-2.72) | 1.1 (0.43-2.79) | 3.85 (0.89-16.69) |
| Limited cutaneous SSc | 0.76 (0.27-2.15) | 0.82 (0.37-1.81) | 0.85 (0.32-2.24) | 1.21 (0.51-2.90) | 0.57 (0.17-1.95) |
| ACA | 1.20 (0.41-3.53) | 1.58 (0.69-3.58) | 1.24 (0.49-3.21) | 0.76 (0.32-1.79) | 0.14 (0.04-0.53) |
| Scl70 antibodies | 0.36 (0.10-1.30) | 0.61 (0.25-1.47) | 0.71 (0.24-2.05) | 1.24 (0.50-3.08) | 3.95 (0.94-16.58) |
| VAS general > median | 0.59 (0.21-1.68) | 0.70 (0.32-1.54) | 1.70 (0.68-4.23) | 1.47 (0.65-3.30) | 1.61 (0.61-4.24) |
| MRSS > median | 0.75 (0.25-2.29) | 0.83 (0.38-1.84) | 0.69 (0.25-1.88) | 0.87 (0.36-2.08) | 2.26 (0.69-7.42) |
| VAS digital > median | 1.31 (0.46-3.71) | 1.41 (0.64-3.10) | 0.58 (0.22-1.50) | 0.54 (0.23-1.26) | 0.98 (0.35-2.72) |
| Digital ulceration | 0.84 (0.29-2.38) | 0.75 (0.32-1.46) | 0.62 (0.23-1.64) | 0.48 (0.20-1.15) | 0.98 (0.31-3.15) |
| Finger-skin thickening | 3.64 (1.20-11.0) | 2.91 (1.27-6.65) | 0.32 (0.11-0.94) | 0.44 (0.18-1.09) | 0.38 (0.14-1.02) |
| VAS Raynaud > median | 0.74 (0.26-2.09) | 1.27 (0.57-2.81) | 1.07 (0.44-2.60) | 0.81 (0.36-1.81) | 0.78 (0.29-2.06) |
| Digestive symptoms | 0.82 (0.16-4.09) | 2.12 (0.69-6.51) | 0.66 (0.21-2.14) | 0.78 (0.24-2.51) | 0.23 (0.05-0.89) |
| VAS digestive> median | 0.22 (0.05-0.82) | 0.48 (0.24-1.23) | 0.71 (0.29-1.71) | 1.27 (0.56-2.84) | 1.51 (0.52-4.39) |
| VAS pulmonary> median | 0.85 (0.30-2.40) | 1.07 (0.52-2.41) | 0.51 (0.21-1.26) | 0.76 (0.34-1.69) | 0.75 (0.29-1.96) |
| Synovitis | - | - | 2.51 (0.44-10.64) | - | - |
| Disability |  |  |  |  |  |
| HAQ-DI > median | 0.37 (0.12-1.34) | 0.69 (0.31-1.52) | 0.65 (0.26-1.58) | 1.05 (0.47-2.32) | 0.89 (0.34-2.34) |
| Cochin scale > median | 0.59 (0.03-13.40) | 0.74 (0.22-2.47) | NA | 9.77 (0.01-100) | 1.36 (0.53-3.47) |
| ** Standardized alcoholic beverage = 12g/glass  * Neurological disease (central or peripheral) that result in palsy; ^†^ Known urethral stricture, benign prostatic hyperplasia, prostatic cancer, prolapse (uterus, rectum or bladder), or bladder cancer in the past; ^‡^ Medical drugs with known urinary side effect (eg: tricyclic antidepressant, antipsychotic, antiparkinsonians, muscle relaxant, antihistamines, antispasmodic).  ACA: Anti-centromere antibodies; Scl70: an antibodies directed against topoisomerase; HAQ-DI: Health assessment questionnaire-Disability Index; y: year; SSc: systemic sclerosis; IQR: interquartile range; MRSS: modified Rodnan skin score; 6MWT: 6-minutes walking test | | | | | |

| **Table 2**: Factors associated with different scenario for patients with and without urinary incontinence. Results are odds ratios from mixed-effect logistic regression. | | | | | |
| --- | --- | --- | --- | --- | --- |
|  | **UI type** | | **UI severity** | | |
|  | **SUI** | **UUI** | **At least monthly UI** | **At least weekly UI** | **At least daily UI** |
|  | 74 / 190 (38.9%) | 67/193 (34.7%) | 91 / 188 (48.4%) | 56 / 188 (29.8%) | 18 / 188 (9.6%) |
| General |  |  |  |  |  |
| Age > median | 1.72 (0.36-8.23) | 9.52 (1.52-59.61) | 6.42 (1.01-37.46) | 11.37 (1.40-92.45) | 4.89 (1.24-16.26) |
| Men | 0.01 (0.001-0.35) | 0.06 (0.01-1.80) | 0.03 (0.01-1.22) | 0.36 (0.02-7.86) | 1.11 (0.23-5.25) |
| BMI Q1 vs Q2  Q3  Q4 | 1.62 (0.20-14.65)  3.07 (0.37-25.54)  30.67 (2.91-322) | 3.46 (0.40-30.12)  7.05 (0.76-65.54)  1.12 (0.13-9.78) | 2.37 (0.24-23.67)  3.12 (0.30-32.19)  10.47 (0.96-113) | - | - |
| Birth-natural route 0 vs 1  2  3 | 1.62 (0.14-19.39)  1.99 (0.25-16.14)  4.86 (0.28-83.97) | 1.97 (0.18-20.88)  6.53 (0.84-50.80)  33.44 (2.15-510) | 1.55 (0.10-22.85)  5.42 (0.53-54.86)  23.75 (0.91-622) | 1.58 (0.10-23.74)  6.31 (0.65-60.71)  10.69 (0.60-191) | 2.02 (0.47-8.83)  0.93 (0.22-3.90)  1.34 (0.22-7.94) |
| Cesarienne 0 vs 1  2 | 3.66 (0.25-53.49)  0.43 (0.02-11.40) | 0.50 (0.04-6.45)  0.08 (0.01-2.38) | 1.09 (0.05-22.67)  0.15 (0.01-6.22) | 0.28 (0.02-4.50)  0.03 (0.01-1.58) | 0.63 (0.07-5.50)  - |
| Pulmonary disease | 0.50 (0.09-2.82) | 0.39 (0.07-2.17) | 0.29 (0.04-2.25) | 1.01 (0.16-6.23) | 1.12 (0.41-3.03) |
| Lung fibrosis | 0.44 (0.09-2.03) | 0.24 (0.05-1.10) | 0.21 (0.04-1.17) | 0.41 (0.08-2.06) | 0.42 (0.12-1.32) |
| Pulmonary hypertension | 0.27 (0.01-4.61) | 4.77 (0.31-72.98) | 0.66 (0.02-19.15) | 4.90 (0.24-97.91) | 2.04 (0.53-7.85) |
| Cardiac disease | 0.72 (0.07-7.34) | 2.80 (0.29-26.92) | 1.15 (0.07-18.07) | 2.01 (0.17-23.63) | 0.37 (0.05-2.88) |
| Heart failure | 0.17 (0.01-43.66) | 75.78 (0.45-129) | 8.68 (0.01-642) | 31.26 (0.09-808) | - |
| Diabetes | 3.09 (0.09-101) | 4.98 (0.19-133) | 5.58 (0.09-340) | 3.25 (0.10-109) | - |
| Neurological palsy* | 2.14 (0.02-222) | 0.22 (0.07-563) | 31.97 (0.05-200) | 7.20 (0.06-820) | 6.86 (1.06-43.96) |
| UI related |  |  |  |  |  |
| Urological disease^†^ | 0.07 (0.01-8.04) | 1.13 (0.03-45.80 | 0.24 (0.01-23.30) | 2.78 (0.05-151.84) | 5.40 (1.22-23.80) |
| Previous stress UI | 19.11 (8.78-41.61) | 3.73 (1.08-12.92) | 11.73 (4.69-29.29) | 6.09 (1.71-21.68) | 11.78 (1.14-121.52) |
| Previous urge UI | 8.42 (2.26-31.35) | 18.57 (8.51-40.56) | 21.33 (7.07-64.27) | 27.57 (11.66-65.15) | 113.56 (4.45-2920) |
| Previous monthly  Weekly  Daily | 14.8 (5.31-41.27)  9.53 (3.31-27.46)  17.87 (4.49-71.05) | 2.63 (1.02-6.79)  34.98 (12.22-100)  23.67 (7.09-79.25) | 9.94 (4.26-23.18)  31.89 (10.57-96.22)  29.14 (7.52-113) | 3.43 (1.13-10.36)  30.30 (10.17-90.26)  53.33 (13.49-210.05) | - |
| Previous fecal incontinence | 2.94 (0.41-20.90) | 2.85 (0.48-16.84) | 1.87 (0.20-17.43) | 4.35 (0.58-32.30) | - |
| Medication |  |  |  |  |  |
| No alcohol vs 1 glass/ day**  2 or more glass/ day | 2.26 (0.33-15.36)  0.93 (0.09-9.84) | 1.00 (0.15-6.62)  3.11 (0.28-34.32) | 3.32 (0.32-34.43)  1.44 (0.09-23.58) | 1.84 (0.23-14.19)  2.30 (0.18-30.09) | 1.09 (0.28-4.26)  3.00 (0.83-10.89) |
| Coffee 0-1cup/d vs 1-2 cup/d  2-3 cup/d  > 3 cup/d | 0.45 (0.07-2.94)  0.45 (0.04-5.05)  0.10 (0.01-5.53) | 0.63 (0.13-3.16)  0.46 (0.22-3.85)  1.06 (0.06-18.85 | 0.57 (0.08-3.90)  0.35 (0.03-4.11)  0.26 (0.07-8.89) | 0.94 (0.14-6.37)  0.73 (0.06-8.19)  1.25 (0.05-34.45) | 0.78 (0.21-2.71)  1.01 (0.24-4.89)  - |
| Corticoids | 0.60 (0.13-2.69) | 0.69 (0.16-3.01) | 0.53 (0.09-3.00) | 0.32 (0.06-1.67) | 0.87 (0.33-2.32) |
| Diuretics | 0.38 (0.06-2.45) | 1.21 (0.23-6.28) | 0.58 (0.08-4.39) | 3.41 (0.71-16.48) | 45.64 (1.17-1776) |
| Opioids | 2.16 (0.25-18.09) | 1.36 (0.19-9.73) | 1.70 (0.15-19.21) | 1.30 (0.15-11.27) | 2.44 (0.72-8.25) |
| Side effect^‡^ | 1.32 (0.11-15.94) | 1.17 (0.13-10.15) | 0.69 (0.05-10.46) | 0.66 (0.10-4.18) | 0.97 (0.11-8.37) |
| Systemic sclerosis |  |  |  |  |  |
| Disease duration >16y | 0.58 (0.11-2.99) | 0.79 (0.15-4.04) | 0.61 (0.09-2.27) | 0.45 (0.07-2.94) | 1.09 (0.39-3.05) |
| Non-raynaud symptoms > 12y | 1.32 (0.23-7.50) | 0.53 (0.10-2.78) | 1.15 (0.17-7.72) | 0.35 (0.01-2.41) | 1.00 (0.35-2.90) |
| Diffuse cutaneous SSc | 0.16 (0.02-1.10) | 0.13 (0.01-1.10) | 0.12 (0.02-0.91) | 0.11 (0.01-1.44) | 0.07 (0.04-1.21) |
| Limited cutaneous SSc | 4.29 (0.77-23.83) | 3.25 (0.54-19.47) | 4.28 (0.72-25.41) | 3.33 (0.40-27.62) | 19.87 (0.85-462) |
| ACA | 4.21 (0.80-22.10) | 16.91 (2.47-115.8) | 9.13 (1.63-51.17) | 25.00 (1.94-322) | 22.32 (1.30-382) |
| Scl70 antibodies | 0.68 (0.12-3.89) | 0.11 (0.02-0.73) | 0.23 (0.04-1.51) | 0.05 (0.01-0.85) | 0.08 (0.05-1.19) |
| VAS general > median | 0.64 (0.16-2.50) | 1.05 (0.28-3.94) | 0.35 (0.07-1.72) | 0.78 (0.17-3.47) | 0.96 (0.35-2.62) |
| MRSS> median | 0.98 (0.21-4.63) | 0.36 (0.07-1.76) | 0.35 (0.06-1.93) | 0.50 (0.08-3.24) | 0.42 (0.14-1.26) |
| VAS digital > median | 1.05 (0.24-4.52) | 1.45 (0.36-5.83) | 1.20 (0.22-6.57) | 2.31 (0.45-11.66) | 0.53 (0.19-1.51) |
| Digital ulceration | 0.67 (0.14-3.25) | 0.77 (0.17-3.43) | 0.39 (0.07-2.27) | 0.55 (0.08-3.51) | 2.07 (0.70-6.18) |
| Finger-skin thickening | 2.25 (0.70-7.23) | 3.69 (0.97-14.11) | 2.71 (0.77-9.53) | 1.40 (0.35-5.61) | 1.83 (0.67-5.01) |
| VAS Raynaud > median | 1.15 (0.30-4.35) | 1.77 (0.49-6.40) | 1.06 (0.24-4.74) | 1.25 (0.29-5.35) | 2.57 (0.55-4.44) |
| Digestive symptoms | 4.91 (0.69-35.03) | 32.38 (3.27-320) | 8.07 (1.24-52.58) | 7.41 (0.58-93.59) | - |
| VAS digestive > median | 1.02 (0.25-4.09) | 2.05 (0.54-7.86) | 0.77 (0.16-3.83) | 1.17 (0.28-4.87) | 2.71 (0.91-8.03) |
| VAS pulmonary > median | 1.60 (0.43-5.97) | 2.76 (0.77-9.92) | 2.07 (0.45-9.47) | 3.54 (0.89-14.10) | 2.42 (0.81-7.20) |
| Synovitis | 0.02 (0.01-1.99) | 0.08 (0.01-2.60) | 0.01 (0.001-0.51) | - | - |
| Disability |  |  |  |  |  |
| HAQ-DI> median | 4.59 (0.99-21.41) | 3.30 (0.90-12.03) | 2.10 (0.46-9.57) | 2.52 (0.65-9.80) | 2.52 (0.85-7.47) |
| Cochin scale> median | 0.90 (0.16-4.97) | 1.64 (0.64-4.23) | 0.90 (0.35-2.35) | 2.89 (0.06-135) | 1.22 (0.31-4.71) |
| * Neurological disease (central or peripheral) that result in palsy; ^†^ Known urethral stricture, benign prostatic hyperplasia, prostatic cancer, prolapse (uterus, rectum or bladder), or bladder cancer in the past; ^‡^ Medical drugs with known urinary side effect (eg: tricyclic antidepressant, antipsychotic, antiparkinsonians, muscle relaxant, antihistamines, antispasmodic).  ACA: Anti-centromere antibodies; Scl70: an antibodies directed against topoisomerase; HAQ-DI: Health assessment questionnaire-Disability Index; y: year; SSc: systemic sclerosis; IQR: interquartile range; MRSS: modified Rodnan skin score; 6MWT: 6-minutes walking test  VAS: visual analogic scale | | | | | |

| **Table 3:** Quality of life (QoL) and evolution of disability between first and third study visits by the presence of urinary incontinence at inclusion. | | | | | | | | | |
| --- | --- | --- | --- | --- | --- | --- | --- | --- | --- |
|  | **Patients continent for urine** (36) | | | | **Patients suffering from UI** (50) | | | | ***P* value^b^** |
|  | **Inclusion** | **Third visit** | **Diff. median (IQR)** | ***P* Value^a^** | **Inclusion** | **Third visit** | **Diff. median (IQR)** | ***P* value^a^** |  |
| **SF-36 domains** |  |  |  |  |  |  |  |  |  |
| Physical functioning | 70 (50–95) | 62.5 (50–85) | 0 (-5-10) | 0.552 | 60 (40–80) | 50 (30–75) | 5 (-5–20) | 0.015 | 0.229 |
| Role physical | 75 (25–100) | 50 (0–100) | 0 (0–25) | 0.216 | 25 (0–75) | 25 (0–75) | 0 (-25–25) | 0.921 | 0.097 |
| Bodily pain | 52 (41–100) | 52 (41–84) | 0 (-10.5–21) | 0.567 | 41 (32–74) | 52 (32–74) | 0 (-16–10) | 0.977 | 0.699 |
| General health | 38.5 (30–57) | 35 (20–51) | 0 (-5–10) | 0.433 | 37.5 (25–47) | 40 (25–55) | 0 (-10–10) | 0.820 | 0.585 |
| Vitality | 55 (45–75) | 55 (35–70) | 0 (-5–15) | 0.587 | 45 (30–60) | 45 (30–55) | 5 (-15–15) | 0.516 | 0.920 |
| Social functioning | 60 (40–70) | 50 (40–70) | 0 (0–20) | 0.108 | 60 (40–70) | 50 (40–70) | 0 (-10–10) | 0.899 | 0.217 |
| Mental health | 68 (60–72) | 68 (60–76) | 0 (0–8) | 0.072 | 68 (56–72) | 64 (52–72) | 0 (-4–12) | 0.299 | 0.765 |
| Role emotional | 100 (33.3–100) | 100 (0–100) | 0 (0–0) | 0.357 | 66.7 (0–100) | 66.7 (0–100) | 0 (-33–33) | 0.811 | 0.671 |
| **SF-36 component summary** |  |  |  |  |  |  |  |  |  |
| Physical | 44.3 (33.8–51.6) | 38.2 (27.4–46.7) | 0.1 (-1.6–9.4) | 0.157 | 37.3 (26.9v43.8) | 33.9 (28.7–42.9) | -0.3 (-3.8–6.7) | 0.999 | 0.263 |
| Mental | 48.2 (41.5–51.1) | 47.3 (35.7–52.6) | 0.5 (-3.3–4.7) | 0.637 | 47.8 (38.7–52.0) | 46.7 (38.7–52.0) | 0.1 (-4.2–2.6) | 0.706 | 0.475 |
| **Iqol** |  |  |  |  |  |  |  |  |  |
| Score | 100 (98.2–100) | 100 (96.8–100) | 0 (0–2.3) | 0.129 | 92.7 (81.4–98.2)^†^ | 93.6 (80.9–99.1) | 0.9 (-3.6–8.2) | 0.234 | 0.963 |
| **Disability** |  |  |  |  |  |  |  |  |  |
| HAQ-DI | 0.375 (0–1) | 0.375 (0–1.125) | -.125 (-.25–0) | 0.006 | 0.75 (0.25–1.25) | 0.5 (0.375–1.375) | 0 (-.25-.187) | 0.541 | 0.190 |
| Cochin hand scale | 3.3 (0–31.1) | 8.9 (1.1–22.2) | -0.6 (-5.6–0) | 0.076 | 4.4 (1.1–18.9) | 6.7 (1.1–16.7) | 0 (-6.7-1.1) | 0.164 | 0.842 |
| A) comparison between first and third visits; b) comparison between patients with and without UI  Iqol: Incontinence quality of life questionnaire; SF-36 MCS; the 36-item Short-Form health survey, Mental Component Summary; SF-36 PCS: the 36-item Short-Form health survey, Physical Component Summary; UI: urinary incontinence | | | | | | | | | |
